# Retinal artery to vein ratio is associated with cerebral microbleeds in individuals with type 1 diabetes

**DOI:** 10.1097/HJH.0000000000003690

**Published:** 2024-02-27

**Authors:** Aleksi Tarkkonen, Ward Fickweiler, Marika Eriksson, Jennifer K. Sun, Lena M. Thorn, Paula Summanen, Per-Henrik Groop, Jukka Putaala, Juha Martola, Daniel Gordin

**Affiliations:** a HUS Medical Imaging Center, Radiology, University of Helsinki and Helsinki University Hospital; b Folkhälsan Institute of Genetics, Folkhälsan Research Center; c Department of Nephrology, University of Helsinki and Helsinki University Hospital; d Research Program for Clinical and Molecular Metabolism, Faculty of Medicine, University of Helsinki; e Neurology, University of Helsinki and Helsinki University Hospital, Helsinki, Finland; f Department of Ophthalmology, Harvard Medical School, Boston, Massachusetts, USA; g Department of Diabetes, Central Clinical School, Monash University, Melbourne, Australia; h Minerva Foundation Institute for Medical Research; i Department of General Practice and Primary Healthcare, University of Helsinki and Helsinki University Hospital, Helsinki, Finland; j Beetham Eye Institute, Joslin Diabetes Center, Harvard Medical School, Boston, Massachusetts, USA; k Department of Ophthalmology, University of Helsinki and Helsinki University Hospital, Helsinki, Finland

**Keywords:** cerebral microbleeds, cerebrovascular disease, diabetic retinopathy, retinal vessel parameters, type 1 diabetes

## Abstract

**Objectives:**

A third of asymptomatic individuals with type 1 diabetes (T1D) show signs of cerebrovascular disease in brain MRI. These signs associate with advanced stages of diabetic retinal disease, but not in mild or moderate retinopathy. We aimed to evaluate a wider spectrum of retinal changes by exploring the relationship between quantitative measures of retinal vessel parameters (RVP) and cerebrovascular changes in T1D.

**Methods:**

We included 146 neurologically asymptomatic individuals with T1D [51% women, median age 40 (33.0–45.1) years] and 24 healthy, sex-matched and age-matched controls. All individuals underwent a clinical and biochemical work-up and brain MRI, which was evaluated for cerebral microbleeds (CMBs), white matter hyperintensities, and lacunar infarcts. RVPs, including central retinal arteriole (CRAE) and central retinal vein (CRVE) equivalents and the ratio of the two variables (arteriovenous ratio, AVR) were assessed quantitatively by a computer-assisted method (IVAN software, version 3.2.6) from fundus images.

**Results:**

Among T1D participants, those with CMBs had a lower arteriovenous ratio (AVR) compared with those without CMBs (*P* = 0.023). AVR was inversely associated with the amount of CMBs (*r* = −0.063, *P* = 0.035). CMB prevalence was higher in those with AVR below the median (31%) compared with above the median (16%, *P* < 0.001), and this difference was significant also after individuals with only no-to-mild retinopathy were included (28 vs. 16%, *P* = 0.005). A correlation between blood pressure and CRAE (*r* = −0.19, *P* = 0.025) appeared among those with T1D.

**Conclusion:**

Regardless of the severity of diabetic retinopathy, AVR is associated with the existence of CMBs in T1D.

## INTRODUCTION

The retina is a structure of the eye where characteristics of the microvasculature can noninvasively be viewed. Microaneurysms, hemorrhages, arteriolar-venular nicking, and arteriolar narrowing are findings in the retinal microcirculation in individuals with hypertension and diabetes [1]. Multiple studies suggest that these abnormalities of the retinal vasculature reflect pathology of the systemic microvasculature. This pathology, in turn, predicts cardiovascular morbidity and mortality [2–4]. We recently discovered that cerebral small vessel disease (SVD), particularly cerebral microbleeds (CMBs), is more common in young people with type 1 diabetes without any signs of neurological disease than in healthy controls [5]. In contrast, the differences between patients with type 1 diabetes and healthy controls in ischemic changes such as white matter hyperintensities and lacunar infarcts were not significant [5]. In the same cohort, cerebral SVD was associated with the severity of diabetic retinal disease (DRD), assessed with a validated clinical grading [6,7]. Importantly, the association was true only in those with either moderate or the most severe, sight-threatening form of DRD, proliferative diabetic retinopathy.

These findings raised a hypothesis about whether cerebral SVD is associated with other retinal changes measured as retinal vessel parameters (RVP) by Integrative Vessel Analysis (IVAN) (University of Wisconsin-Madison, Wisconsin, USA). This software uses digital retinal imaging technology to semi-automatically assess RVP. Through this method, quantification of vascular caliber is available, which can reveal both arteriolar narrowing and venular widening [8,9]. The retinal arteriole-to-venule ratio (AVR) has been shown to correlate with the risk of stroke and coronary artery disease [10]. Furthermore, the software has good internal agreement and reliability [9,11].

Our first aim was to assess whether RVP changes correlated with CMBs, which are the most common form of SVD in patients with type 1 diabetes. Second, we studied whether diabetes-related covariates, including glycemic control, blood pressure, and the presence of other diabetic microvascular complications, were associated with RVP. Third, we compared RVP between individuals with type 1 diabetes and healthy controls.

## METHODS

This study is a part of the prospective nationwide Finnish Diabetic Nephropathy (FinnDiane) Study. The design of the FinnDiane study has been described elsewhere in more detail [12]. For this substudy, 191 subjects with type 1 diabetes were examined between 2011 and 2017 [5]. A total of 30 heathy age-matched and sex-matched controls were also enrolled. The participants were adults less than 50 years of age. None of the participants had a history or clinical manifestations of any neurological disease. However, one individuals who turned out to have experienced previous neurosurgery and two participants showing findings consistent with multiple sclerosis were excluded from the analysis. Exclusion criteria were kidney failure with replacement therapy, coronary heart disease or peripheral arterial disease, and contraindications for MRI. The study was carried out in accordance with the Declaration of Helsinki, and the protocol was approved by the Ethics Committee of the Helsinki and Uusimaa Hospital District (ID: HUS/2184/2017). Each participant gave their written informed consent prior to participation.

All participants underwent a clinical evaluation and comprehensive laboratory work-up at the FinnDiane research unit at Biomedicum Helsinki, Finland. The visit included a review of previous medical history, office blood pressure measurement and structured questions on lifestyle and anthropometrics. Blood pressure was measured twice with 2 min intervals in the sitting position after a 10 min rest, mean value of these two measurements was used as a continuous variable. Blood samples were drawn to analyze concentrations of plasma creatinine, lipids and lipoproteins [total cholesterol, low-density lipoprotein (LDL), high-density lipoprotein (HDL) and triglycerides], high-sensitivity C-reactive protein (hs-CRP), and blood glycated hemoglobin (HbA1c) using standardized methods. Diabetic kidney disease was defined as increased albumin excretion rate (≥200 μg/min or ≥300 mg/24 h) in two out of three urine collections. Glomerular filtration rate (eGFR) was estimated by the CKD-EPI-formula [13,14].

The Early Treatment of Diabetic Retinopathy (ETDRS) scale was used to classify diabetic retinopathy severity [15]. Acquisition, evaluation and grading of retinal images has been described elsewhere [7]. Tropicamide was used for pupil dilatation [7]. For analysis, participants were categorized based on diabetic retinopathy severity: ETDRS score 35 or less indicated no-to-mild nonproliferative diabetic retinopathy (NPDR), 43–57 moderate to severe NPDR, and ETDRS score at least 61 proliferative diabetic retinopathy (PDR). Presence of any diabetic retinopathy was defined as ETDRS score at least 20 [7].

Carotid ultrasound was performed on the left and right carotid arteries. The distal 1 cm segment of the common carotid artery segment, immediately before the origin of the bulb was scanned using an ultrasound scanner equipped with a 10 MHz linear probe (MyLab 70, Esaote, Genova, Italy) and implemented with a radiofrequency-based tracking of arterial wall (QIMT) that allows an automatic and real-time determination of far-wall carotid intima–media thickness (CIMT). The mean of two measurements of the left and right CIMT was calculated for the subsequent analyses. To calculate central pulse wave velocity (cPWV), the path lengths from the sternal notch to the femoral pulse were measured.

Within 1 year of the clinical and laboratory work-up, all participants underwent a 3T brain MRI (Achieva, Philips, Best, The Netherlands) at the Medical Imaging Center at the Helsinki University Hospital. MR-sequences used were T1, T2, FLAIR, T1 MPRAGE, DWI, SWI, T2∗ and MRA TOF. Small-vessel disease (SVD) consisting of CMBs, white matter hyperintensities (Fazekas scale, with category ≥1 considered a significant burden), or lacunar infarcts were evaluated according to standardized criteria [16]. The number of CMBs were calculated and analyzed as a continuous variable. The MRI evaluation was performed by a senior neuro-radiologist (J.M.) with more than 10 years of experience, blinded to clinical and laboratory parameters.

**FIGURE 1:**
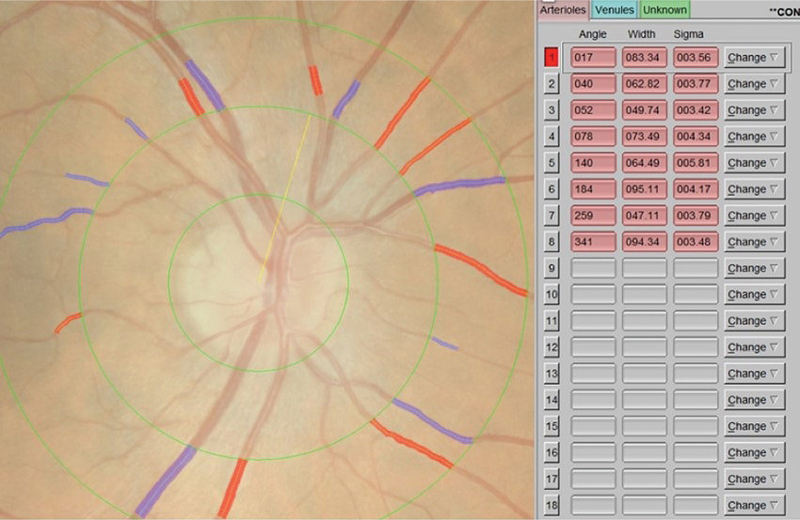
Screenshot of Integrative Vessel Analysis software interface. Red vessels manually identified as arterioles and blue vessels as veins.

RVP indices were calculated using a computer-assisted semi-automated imaging software [Integrative Vessel Analysis (IVAN) software, version 3.2.6, University of Wisconsin, Madison, Wisconsin, USA] from digitized photographs. A standardized protocol at Centre for Eye Research Australia was followed [17]. The software identifies six largest arterioles and venules passing completely through a circumferential zone 0.5–1 disc diameter from the optic disc margin [18]. The software identifies the optic disc and measures the diameters of individual vessels. The grader confirms the correctness of vessel type selected by the program. Three indices are calculated by IVAN software, central retinal arteriole (CRAE) and central retinal vein (CRVE) equivalents and the ratio of the two variables (arteriovenous ratio, AVR), using the Knudtson's revision of the Parr–Hubbard formula [19]. CRAE was divided by CRVE to obtain the AVR. The grader (A.T.) was masked to subject identity and treatment. The right eye's image was primarily analyzed, unless unsuitable due to poor image quality leading to hazy or obscured views of the retinal vasculature (Fig. 1). In these cases of ungradable right eye images, the left eye's image was graded for RVP indices (Supplemental Data File).

### Statistical analysis

The normality of distribution of CRAE, CRVE, and AVR was evaluated using the Shapiro–Wilks test. Two-sided Student's *t* test was used to compare normally distributed variables between groups, whereas nonnormally distributed variables were analyzed with the Mann–Whitney *U* test. Data are presented as mean ± SD for normally distributed variables and median with interquartile range (IQR) for nonnormally distributed variables.

In individuals with diabetes, we analyzed the correlation between RVPs and clinical and laboratory variables using the Spearman's correlation test and linear regression analysis. Standardized coefficients were calculated to evaluate how many standard deviations RVPs would change per standard deviation increase in a specific clinical variable.

We also used binary logistic regression models to identify covariates that were independently associated with CMBs. Results are presented as odds ratios (ORs) with 95% confidence intervals (CIs). Along with RVP, HbA1c and SBP were included in the logistic regression analysis because of their associations with RVP. We conducted a sensitivity analysis by excluding individuals with at least moderate retinopathy (ETDRS >35) to assess the associations in a population of individuals with none-to-mild retinopathy.

Finally, we divided the subjects based on the median AVR into two groups: those with AVR more or less than the median value. We compared the clinical parameters of these groups using the Mann–Whitney *U* test and the prevalence of CMBs, WMHs, and lacunes using the chi-squared test.

Statistical analyses were performed using R (version 4.0.2). A two-sided *P* less than 0.05 was set as the threshold for statistical significance.

## RESULTS

RVP measurements were of sufficient quality in 77.7% (146/188) of individuals with type 1 diabetes and 80% (24/30) of the healthy control subjects. No significant differences in clinical characteristics were observed between individuals with type 1 diabetes who were included or excluded from the final analysis (*P* > 0.05 in all). There was no substantial difference in clinical characteristics between healthy controls who were included or excluded from the final analysis.

Of a total of 170 individuals included in whom RVPs were measured, 39% (57/146) of individuals with type 1 diabetes and 8.3% (2/24) of controls had cerebral SVD. Seventy percent of individuals with type 1 diabetes had no or mild diabetic retinopathy, 16% had moderate or severe retinopathy, and 14% had PDR. None of the controls showed signs of DRD.

In individuals with type 1 diabetes, diabetes duration was 21.7 (18.2–30.8) years and HbA1c 66 ± 13 mmol/mol. SBP was 129 ± 15 mmHg, total cholesterol 4.4 (4.0–5.0) mmol/l, LDL 2.37 (2.00–2.92) mmol/l, HDL 1.51 (1.25–1.80) mmol/l and hs-CRP 1.11 (0.50–2.31) mg/l. A total of 85 (58%) of subjects had either SBP over 130 mmHg or DBP over 80. Twenty-four-hour urinary albumin excretion rate was 13.7 (10.3–18.0)  mg. Increased urinary albumin rate was present in 20 of 146 (13.7%) of subjects. In the T1D group, 34 (23%) were on statins, 55 (38%) on antihypertensive medication and 12 (8.2%) on low-dose aspirin. We lack granular details about the exact antihypertensive drugs; 30 subjects were on one type, 16 were on two types, 7 were on three, and 1 individual was on four different antihypertensive medications. The most frequently prescribed classes were angiotensin-converting enzyme inhibitors or angiotensin receptor blockers, followed by calcium channel blockers, and then beta-receptor blockers. None of the age-matched and sex-matched controls had any of these medications. A total of 85 (58%) in the T1D group had either SBP greater than 130 mmHg or DBP greater than 80 mmHg (Table 1).

**TABLE 1:** Participant characteristics

	People with type 1 diabetes (*n* = 146)	Control subjects (*n* = 24)	*P*
Female sex [*n* (%)]	74 (51)	14 (58)	0.626
Age (years)	40.6 (33.7–45.6)	37.7 (31.1–41.1)	0.433
Diabetes duration (years)	21.7 (18.2–30.8)	–	–
BMI (kg/m^2^)	26.9 ± 4.2	24.7 ± 3.3	0.002
SBP (mmHg)	131 ± 14	122 ± 11	0.002
DBP (mmHg)	77 (72–82)	76 (74–85)	0.384
HbA_1c_ (%) (mmol/mol)	8.2 ± 1.2 (66 ± 13)	5.3 ± 0.2 (32 ± 2)	<0.001
Creatinine (μmol/l)	68 (61–80)	74 (66–80)	0.305
Total cholesterol (mmol/l)	4.4 (4.0–5.0)	4.6 (4.2–5.4)	0.184
LDL cholesterol (mmol/l)	2.4 (2.0–2.9)	2.6 (2.4–3.3)	0.029
HDL cholesterol (mmol/l)	1.51 (1.25–1.80)	1.46 (1.28–1.62)	0.442
Triglycerides (mmol/l)	0.90 (0.71–1.43)	0.84 (0.71–1.25)	0.540
Statin therapy [*n* (%)]	34 (23)	0 (0)	0.002
Antihypertensive medication [*n* (%)]	55 (38)	0 (0)	<0.001
Aspirin therapy [*n* (%)]	12 (8.2)	0 (0)	0.232
Albuminuria [*n* (%)]	20 (14)	0 (0)	0.055
Retinal photocoagulation [*n* (%)]	32 (22)	0 (0)	0.002
Coronary heart disease [*n* (%)]	1 (0.5)	0 (0)	1.000
Current smoking [*n* (%)]	11 (7.5)	4 (17)	0.118

Clinical characteristics and MRI findings in participants with type 1 diabetes and control subjects matched for age and sex. HDL, high-density lipoprotein; LDL, low-density lipoprotein.

### Retinal vessel parameters between individuals with type 1 diabetes and control subjects

CRAE were normally distributed (*P* = 0.303 in Shapiro–Wilks's test), whereas CRVE and AVR were not (*P* < 0.001 and *P* = 0.036). There were no significant differences in CRAE between people with type 1 diabetes and healthy controls (Table 1).

### Retinal vessel parameters in individuals with type 1 diabetes

CRAE were not different between male and female individuals, but CRVE were larger among female individuals [277 (256–303) μm vs. 300 (272–325) μm, *P* < 0.001]. There was no difference in AVR between male and female individuals. None of the RVP were different in individuals with no-to-mild NPDR vs. those with more severe, similarly the RVP metrics were not different in individuals with moderate-to-severe DRD vs. PDR. CRAE were smaller in individuals with PDR vs. without PDR (*P* = 0.029), whereas CRVE or AVR were not different between eyes with vs. without PDR. CIMT was negatively associated with AVR (*P* = 0.034). None of the RVP indices were associated with diabetic kidney disease (*P* > 0.05). Similarly, no associations between lipids, cPWV, hs-CRP, antihypertensive or lipid-lowering medications and RVP were observed (*P* > 0.05 in all) (Table 2).

**TABLE 2:** Relationship of various characteristics to central retinal arteriolar equivalent; central retinal venular equivalent, and arteriolar-to-venous ratio

	CRAE (μm)	*P*	CRVE (μm)	*P*	AVR	*P*
Male	165.15 (155.02–183.11)	0.100	285.00 (258.97–313.67)	**<0.001**	0.61 (0.56–0.65)	0.789
Female	175.95 (155.85–196.39)	*NA*	300.00 (272.51–325.54)	*NA*	0.61 (0.54–0.66)	*NA*
Age <40 years	176.00 (156.64–195.74)	0.106	295.00 (266.49–317.64)	0.422	0.61 (0.55–0.66)	0.304
Age ≥40 years	164.87 (155.27–183.90)	*NA*	290.42 (258.60–317.94)	*NA*	0.60 (0.54–0.64)	*NA*
HbA_1c_ <65 mmol/mol	171.87 (155.44–193.44)	0.673	287.28 (258.92–309.90)	0.105	0.64 (0.56–0.67)	**0.035**
HbA_1c_ ≥65 mmol/mol	165.84 (271.26–320.00)	*NA*	293.55 (155.75–191.70)	*NA*	0.59 (0.54–0.63)	*NA*
BMI <25 kg/m^2^	171.97 (155.11–195.67)	0.721	292.81 (269.64–318.02)	0.264	0.61 (0.54–0.65)	0.596
BMI ≥25 kg/m^2^	167.40 (155.85–187.58)	*NA*	287.10 (259.23–317.86)	*NA*	0.60 (0.55–0.66)	*NA*
SBP <130 mmHg	172.96 (157.10–195.52)	0.180	295.74 (269.32–318.44)	0.099	0.61 (0.54–0.65)	0.977
SBP ≥130 mmHg	164.94 (154.50–184.94)	*NA*	281.66 (258.57–316.76)	*NA*	0.60 (0.55–0.65)	*NA*
DBP <85 mmHg	171.50 (155.67–194.56)	0.121	292.75 (261.04–318.45)	0.393	0.61 (0.54–0.66)	0.818
DBP ≥85 mmHg	161.01 (155.37–176.46)	*NA*	278.80 (263.10–301.71)	*NA*	0.59 (0.56–0.66)	*NA*
Heart rate <70/min	166.13 (155.11–193.29)	0.607	288.20 (260.76–319.59)	0.963	0.61 (0.53–0.67)	0.857
Heart rate ≥70/min	170.58 (156.87–187.61)	*NA*	292.75 (263.37–316.80)	*NA*	0.60 (0.55–0.65)	*NA*
Nonsmoker^a^	169.24 (155.44–189.38	0.611	288.20 (260.78–318.14	0.082	0.61 (0.54–0.66)	0.226
Smoker^a^	176.31 (156.80–198.00)	*NA*	310.12 (292.70–315.74)	*NA*	0.57 (0.54–0.61)	*NA*
None-to-mild NPDR	171.61 (159.82–191.11)	0.241	291.45 (261.30–314.67)	0.624	0.60 (0.55–0.66)	0.458
Moderate-severe NPDR	167.53 (157.47–201.69)	*NA*	293.14 (273.95–332.87)	*NA*	0.62 (0.567–0.656)	*NA*
PDR	157.54(149.43–173.26)	**0.029**	271.93(247.40–314.71)	0.331	0.58 (0.52–0.64)	0.226
No CMBs	171.10 (155.75–194.23)	*NA*	290.95 (262.72–316.30)	*NA*	0.61 (0.56–0.66)	*NA*
≥1 CMB	163.68 (152.86–180.35)	0.159	292.98 (261.82–318.46)	0.577	0.58 (0.52–0.64)	**0.023**
Fazekas 0/3	171.50 (155.51–191.11)	*NA*	291.98 (262.26–318.43)	*NA*	0.61 (0.54–0.65)	*NA*
Fazekas ≥1/3	165.17 (155.85–189.28)	0.940	290.42 (269.62–301.39)	0.552	0.60 (0.58–0.66)	0.829
Lacunes absent	170.08 (155.44–192.20)	*NA*	291.45 (262.99–318.22)	*NA*	0.61 (0.54–0.66)	*NA*
Lacunes present	158.72 (157.28–160.88)	0.301	259.02 (253.15–276.85)	0.220	0.63 (0.58–0.64)	0.978
WMHs absent	171.97 (155.51–189.48)	*NA*	293.14 (262.26–318.47)	*NA*	0.61 (0.53–0.65)	*NA*
≥1 WMH	164.33 (155.85–197.75)	0.946	288.20 (269.62–302.23)	0.545	0.61 (0.58–0.66)	0.426
Microalbuminuria	164.75 (155.76–176.79)	*NA*	286.59 (258.24–327.30)	*NA*	0.58 (0.54–0.63)	*NA*
No microalbuminuria	170.33 (155.51–192.74)	0.614	291.45 (262.94–316.54)	0.853	0.61 (0.55–0.66)	0.189
Antihypertensive drugs	164.87 (154.70–179.22)	0.128	280.04 (254.61–315.95)	0.273	0.59 (0.54–0.65)	0.278
No antihypertensive drugs	175.39 (156.35–195.35)		293.96 (266.33–317.94)		0.61(0.56–0.66)	
Lipid-lowering medication	164.00	0.250	293.91	0.915	0.58	0.097
No lipid-lowering medication						

AVR, arteriolar-to-venous ratio; CMB, cerebral microbleed; CRAE, central retinal arteriolar equivalent; CRVE, central retinal venular equivalent; DR, diabetic retinopathy; PDR, proliferative diabetic retinopathy; WMH, white-matter hyperintensity.

a Current smoker/nonsmoker values given as median (interquartile range).

CRAE was associated with SBP but not with DBP. Neither CRVE nor AVR were associated with SBP or DBP. CRAE did not correlate with HbA_1c_. However, CRVE correlated positively with HbA_1c_ and AVR negatively. None of the three variables correlated with the duration of diabetes (*P* > 0.05 for all) (Table 3).

**TABLE 3:** Standardized coefficient values of linear regression for different characteristics

	CRAE, *r*	*P*	CRVE, *r*	*P*	AVR, *r*	*P*
Age (years)	−0.154	0.063	0.024	0.776	−0.138	0.096
Duration (years)	−0.050	0.554	0.075	0.377	−0.143	0.089
HbA_1c_ (mmol/mol)	−0.008	0.921	0.210	**0.008**	−0.213	**0.007**
BMI (kg/m^2^)	−0.012	0.885	−0.015	0.856	0.026	0.752
SBP (mmHg)	−0.185	**0.025**	−0.057	0.498	−0.094	0.261
DBP (mmHg)	−0.107	0.215	0.017	0.840	−0.078	0.368
CMBs (*n*)	−0.091	0.220	0.140	0.056	−0.063	**0.035**
WMHs (*n*)	−0.015	0.846	−0.034	0.660	0.002	0.984
Lacunes (*n*)	−0.076	0.368	−0.088	0.326	0.005	0.958
hs-CRP (mg/l)	0.148	0.087	0.031	0.720	0.090	0.298
Total cholesterol (mmol/l)	−0.031	0.704	0.005	0.954	−0.030	0.710
LDL cholesterol (mmol/l)	−0.022	0.781	−0.044	0.577	0.020	0.803
HDL cholesterol (mmol/l)	0.008	0.928	0.089	0.290	−0.064	0.451
Triglycerides (mmol/l)	−0.059	0.464	−0.054	0.501	−0.018	0.822
eGFR (ml/min/1.73m^2^)	0.096	0.242	−0.094	0.252	0.187	**0.022**

Linear regression coefficients values of univariate analysis and *P* for correlation between RVP and various characteristics. AVR, arteriolar-to-venous ratio; CMB, cerebral microbleed; CRAE, central retinal arteriolar equivalent; CRVE, central retinal venular equivalent; eGFR, estimated glomerular filtration rate; HDL, high-density lipoprotein; hs-CRP, high sensitivity C-reactive protein; LDL, low-density lipoprotein; RVP, retinal vessel Parameter; WMH, white matter hyperintensity.

There was no difference in CRAE or CRVE between subjects with or without CMBs. AVR were, however, lower among those with CMBs. There was no correlation between CRAE or CRVE and the number of CMBs, but AVR were associated with the number of CMBs. RVP indices were not associated with WMHs, Fazekas classification or lacunes (*P* > 0.05 for all) (Tables 2 and 3).

**FIGURE 2:**
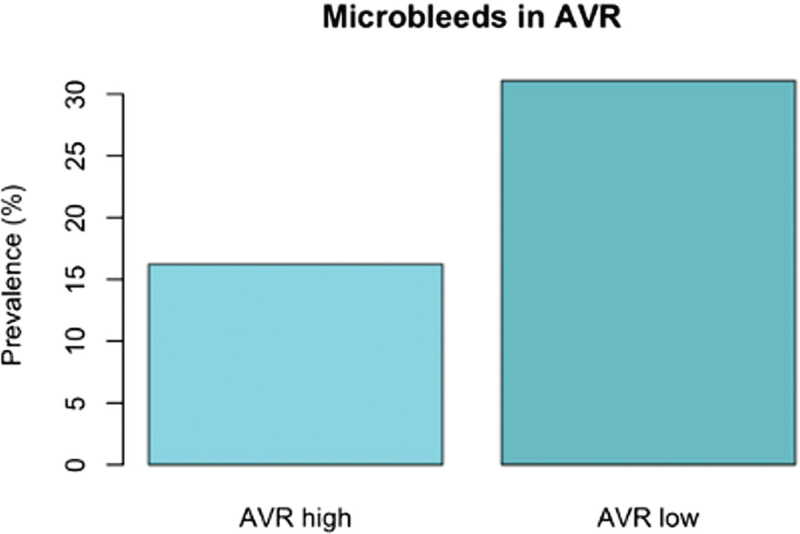
Prevalence of cerebral microbleeds in individuals with type 1 diabetes according to arterial-to-venous ratio less or more than median value 0.610.

**FIGURE 3:**
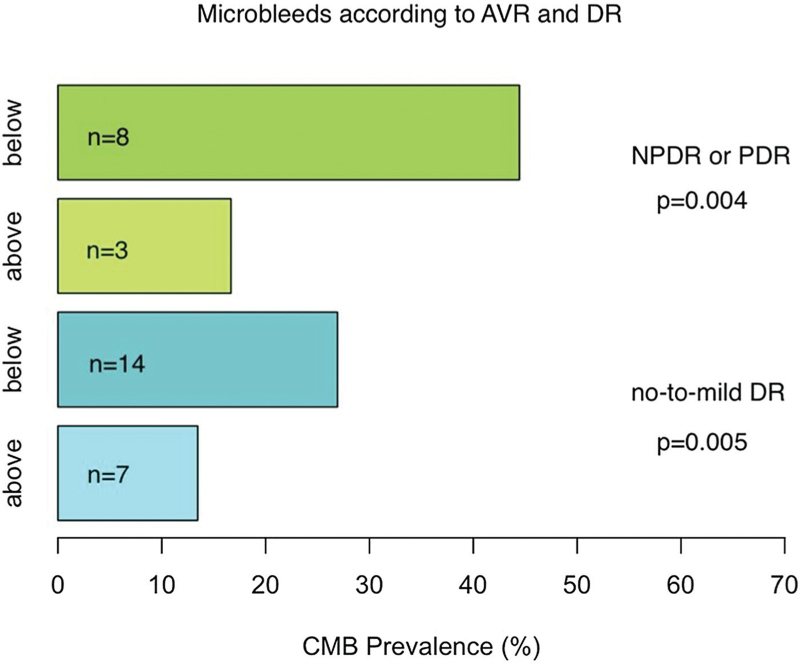
Prevalence of cerebral microbleeds in individuals with type 1 diabetes according to arterial-to-venous ratio above or below median value (0.610) among different levels of retinopathy. On top of each bar is the number of individuals with CMBs. CMBs, cerebral microbleeds.

Finally, we divided individuals with T1D into different subsets between those with AVR below median value or above median value. Here we observed a difference in HbA_1c_ and in CMB prevalence between these two groups, both values being higher in AVR below median group (Table 4 and Fig. 2). After further dividing DRD groups by median values of AVR, we observed a segregation in CMB prevalence (Fig. 3), and this difference was significant among both those with no-to-mild retinopathy and among those with ETDRS greater than 35.

**TABLE 4:** Descriptive statistics

	AVR above median	AVR below median	*P* value
Age (years)	40.06 (33.33–44.82)	40.75 (34.88–46.31)	0.136
Duration (years)	21.74 (18.34–27.27)	21.63 (17.96–32.15)	0.321
HbA_1c_ (mmol/mol)	59.50 (56–70)	67.00 (60.0–75.5)	**0.001**
BMI (kg/m^2+^)	25.20 (24.06–28.78)	26.78 (23.97–29.79)	0.177
SBP (mmHg)	127.25 (119.00–140.25)	130.25 (124.00–140.00)	0.217
DBP (mmHg)	76.50 (72.0–81.5)	77.00 (72.25–82.00)	0.873
CMBs [*n* (%)]	12 (12.10)	23 (27.10)	**0.001**
WMHs [*n* (%)]	18 (21.20)	16 (18.8)	0.975
Lacunes [*n* (%)]	2 (2.34)	1 (1.18)	0.637
hs-CRP (mg/l)	1.06 (0.59–2.89)	1.12 (0.49–2.05)	0.588
Total cholesterol (mmol/l)	4.47 (4.03–5.00)	4.44 (3.97–4.93)	0.592
LDL-cholesterol (mmol/l)	2.46 (2.04–2.89)	2.46 (1.99–2.94)	0.671
HDL-cholesterol (mmol/l)	1.55 (1.30–1.85)	1.44 (1.21–1.77)	0.097
Triglycerides (mmol/l)	0.86 (0.66–1.15)	0.98 (0.75–1.57)	0.069
eGFR (ml/min/1.73 m^2^)	107.65 (97.03–114.92)	105.97 (89.40–113.36)	0.063

People with type 1 diabetes divided into two groups: those with AVR below or above median AVR, which was 0.610 among both people with type 1 diabetes and healthy controls. Categorical variables of people with type 1 diabetes having at least one CMB/WMH/Lacune. *P* for difference between these two groups. Median values with interquartile range of different clinical parameters are shown. AVR, arteriolar-to-venous ratio; CMB, cerebral microbleed; eGFR, estimated glomerular filtration rate; HDL, high-density lipoprotein; hs-CRP, high-sensitivity C-reactive protein; LDL, low-density lipoprotein; WMH, white matter hyperintensity.

In binary logistic regression analysis for the probability of a subject having at least one CMB, this probability was independently associated with AVR in a model including SBP, HbA_1c_, and duration of diabetes [OR 0.428 (95% CI 0.187–0.98), *P* = 0.045]. The association remained even after excluding individuals with moderate-to-severe or proliferative retinopathy (*N* = 37) from the analysis [OR 0.004 (95% CI 0.001–0.863), *P* = 0.0441].

## DISCUSSION

The main finding of our study was that AVR was lower in individuals with type 1 diabetes who also had CMBs in brain MRI. Furthermore, the number of CMBs correlated inversely with the AVR even after adjustment for blood pressure, HbA1c, and duration of diabetes.

We observed previously that people with more severe DRD were more likely to have CMBs [7]. In the present study, we observed that also among groups with no-to-mild DRD or moderate NPDR, CMB prevalence was higher when CRAE or AVR was below median. Furthermore, in logistic regression analysis, there was an association between AVR and existence of CMBs among those subjects without PDR, even after adjustment for SBP and HbA_1c_. These findings suggest that early vascular changes in the retina may reflect cerebral SVD among individuals with type 1 diabetes.

In previous studies, several factors have been observed to affect RVP. Arteriolar caliber has been observed to be influenced by age, race, blood pressure and incident hypertension, obesity, dyslipidemia, and smoking [9,20,21]. Similarly, retinal venular caliber has been observed to be influenced by gender, race, blood pressure, obesity, and smoking [9]. In these studies [9,20,21], however, the number of included individuals were considerably higher compared with our study and none of the studied individuals had type 1 diabetes. Both inter-observer and intra-observer comparisons have been extremely high for RVP assessment [21]. In agreement with earlier results [3,4,22], we found CRAE to be smaller among those with higher blood pressure and CRVE smaller among men. In our study, however, there was no association between blood pressure and CRVE. Neither did we find any associations between RVP and age, antihypertensive medication, albuminuria, BMI, hs-CRP or lipids/lipoproteins, although these associations have been described previously [9,20,21]. However, these previous studies have had larger populations, included subjects over the age of 50 years and did not involve people with type 1 diabetes. Especially, we did not find any relation between albuminuria and RVP (Table 2). In one study, consisting of 5897 participants, an association between CRAE and albuminuria, but not between CRVE and albuminuria, were observed [23].

Arteriolar narrowing is thought to be the most characteristic sign of early hypertensive retinopathy [4]. In our study, CRAE was smaller in individuals with PDR, whereas there was no difference found in CRVE or AVR between eyes with and without PDR. It remains unclear, whether PDR is driving CRAE or the other way around. In multiple studies [2,3,24], arteriolar narrowing has been observed to monotonically and inversely associate with, not only current but also previous BP levels measured years before the retinal photographs were taken. This suggests that reduced CRAE and AVR may reflect cumulative microvascular damage from hypertension over some period. However, some previous results are ambiguous. In one study, both CRAE and CRVE were associated with mean arterial pressure [25]. However, in a later study by the same authors, during a 5-year follow-up, no blood pressure measurements were found to associate with changes in CRAE or CRVE [26].

As retinal arterioles and veins share common anatomic and physiologic features, such as blood–brain barrier and autoregulation of blood pressure, changes in their diameter may reflect similar changes in cerebral arterioles and veins. The association of increase in venular dilation with progression of increased HbA_1c_ was expected and is consistent with data from earlier studies [27]. Increase in venular diameter in eyes with retinopathy is thought to result from retinal hypoxia and from lactate accumulation resulting from hyperglycemia [22,28]. Generalized narrowing of retinal arterioles can be useful in predicting occurrence of new CMBs over time, which warrants further studies. Future studies may also evaluate whether certain thresholds of AVR are highly associated with the presence of CMBs, and if this has management or treatment implications for patients with type 1 diabetes. We did not observe as relation between RVP and small cerebral ischemic changes. We can speculate that WMHs are due to early atherosclerosis and thus strongly associated with age, whereas CMBs seems to be of different cause.

Our study has multiple strengths. The population is relatively large and involves only neurologically asymptomatic individuals with type 1 diabetes and their controls. The distribution of severity of retinopathy based on objective recording of diabetic retinopathy is comprehensive. Nevertheless, some caution must be exercised in interpreting these findings. Measurements were performed by a single masked rater using a standardized method and validated, semi-automated software (IVAN). In this study, only one person (A.T.) rated the fundus photos using the IVAN software. However, the method is validated and semi-automated. W.F., an experienced ophthalmologist, cross-checked 30 measurements with A.T. whereafter they were repeated by A.T. We ended up with very accurate results. Both intra-observer and inter-observer reproducibility were within a 5% difference. In previous literature, both inter-grader and intra-grader measurements show good agreement, which is in line with our accuracy of measurements [29]. In order to assess retinal vasculature, pupils were dilated with tropicamide. Tropicamide has been reported to reduce retinal capillary flow [30]. The effect of tropicamide on retinal vessel dimension was investigated by Frost *et al.*[31]. Only the right eye was dilated whereas the left served as a control. No changes in the vessel width parameters were observed after adjustments for image magnification. Statistical power decreases when subjects are divided into smaller subgroups. An in-depth assessment of blood pressure management could significantly enhance the interpretation of our results. Additionally, the limited availability of robust physical activity data precluded its reliable analysis in this study. Although AVR, CRAE and CRVE, serve as useful metrics in our study, we acknowledge its potential limitations in fully capturing complex retinal vascular changes. Advanced methodologies like optical coherence tomography angiography, scanning laser Doppler flowmetry or adaptive optics, though not employed here because of resource constraints, could provide more precise evaluations in future research. Moreover, the retinal changes that we are measuring here are microscopic. However, computer-assisted standardized protocols of measurement were consistent over time and phenotypic data of this study is thoroughly characterized.

In conclusion, our findings suggest that decreased AVR is associated with the presence of cerebral microvascular disease in individuals with type 1 diabetes, independent of duration of diabetes, glycemic control, blood pressure level, and other traditional risk factors. This assumption needs, however, to be tested in a longitudinal study. Previously, we found an association between CMBs and the severity of retinopathy. The present findings add to this knowledge suggesting there are changes in the retinal vasculature, even among those with no or mild diabetic retinopathy that are indicative of cerebrovascular disorder in middle aged individuals with type 1 diabetes.

## ACKNOWLEDGEMENTS

A.T., D.G., W.F., J.S., J.M., M.I.E., L.T., and P.S. contributed to the study design, acquisition of data, as well as the interpretation of data. A.T. and D.G. had the main responsibility for analyzing the data and writing the first draft of the article. J.M. evaluated the MR images and together with L.T. and D.G. put together the original dataset of the study. M.I.E. and P.S. collected and graded fundus images and designed the ETDRS dataset. D.G., J.P., J.M., M.I.E., L.T., P.S., W.F., J.S. and P.-H.G. critically revised the manuscript. P.-H.G., is the guarantor of this work and, as such, had full access to all the data in the study, and takes responsibility for the integrity of the data and the accuracy of the data analysis.

The skilled technical assistance of Anna Sandelin, Kirsi Uljala, and Jaana Tuomikangas is gratefully acknowledged. We also gratefully thank Pentti Pölönen, Department of Radiology, Helsinki University Hospital for performing the MRI scans. We are indebted to Oili Salonen, Department of Radiology, Helsinki University Hospital for her contribution in planning the MRI protocol. The IVAN software used in the present study was developed by Dr Nicola Ferrier of the University of Wisconsin, Madison School of Engineering and the Department of Ophthalmology and Visual Sciences, University of Wisconsin-Madison, Wisconsin, USA.

Manuscript has not been published previously, but some results of this article were presented at the 2022 annual meeting of European Society of Hypertension in Athens.

Sources of support: the FinnDiane Study was supported by grants from the Folkhälsan Research Foundation, Academy of Finland (316664), Wilhelm and Else Stockmann Foundation, Liv och Hälsa Society, the Medical Society of Finland, Novo Nordisk Foundation (NNF OC0013659), Päivikki and Sakari Sohlberg Foundation, the Finnish Diabetes Research Foundation, and by an EVO governmental grant (TYH2018207). D.G. was supported by Wilhelm and Else Stockmann Foundation, Liv och Hälsa Society, Medical Society of Finland (Finska Läkaresällskapet), Dorothea Olivia, Karl Walter and Jarl Walter Perklén's Foundation, Päivikki and Sakari Sohlberg Foundation, Sigrid Juselius Foundation, Minerva Foundation Institute for Medical Research, the University of Helsinki (Clinical Researcher stint), and the Academy of Finland (UAK1021MRI). None of the funding bodies had any role in the study design, collection, analysis, or interpretation of data. Nor had the funding bodies any role in the writing of the report, nor in the decision to submit the paper for publication.

### Conflicts of interest

A.T. is a shareholder of RokoteNyt Oy. D.G. Lecture or advisory honoraria: AstraZeneca, Bayer, Boehringer Ingelheim, Delta Medical Communications, EASD eLearning, Finnish Nephrology Association, Kidney and Liver Foundation in Finland, and Novo Nordisk. J.M. has received Lecture Honoria Santen. M.I.E. is a shareholder of BCB medical. P.-H.G. has received lecture honoraria from AstraZeneca, Boehringer Ingelheim, Eli Lilly, Elo Water, Genzyme, Medscape, Merck Sharp & Dohme (MSD), Mundipharma, Novartis, Novo Nordisk, PeerVoice, Sanofi, SCIARC, and is an advisory board member of AbbVie, Bayer, Boehringer Ingelheim, Eli Lilly, Janssen, Medscape, MSD, Novartis, Novo Nordisk, and Sanofi. No other potential conflicts of interest relevant to this article were reported.

## Supplementary Material

Supplemental Digital Content
