# Supplementary material for: Retinal artery to vein ratio is associated with cerebral microbleeds in individuals with type 1 diabetes
Source: J Hypertens. 2024 Feb 27;42(6):1039–47. doi: 10.1097/HJH.0000000000003690 (PMC11064917; doi:10.1097/HJH.0000000000003690)

Supplementary Figure 1: Correlation between CRAE and Systolic Blood Pressure This plot illustrates the negative correlation between Central Retinal Arteriolar Equivalent(CRAE) on the y-axis and systolic blood pressure (BP) on the x-axis. Each data pointrepresents a participant in the study.


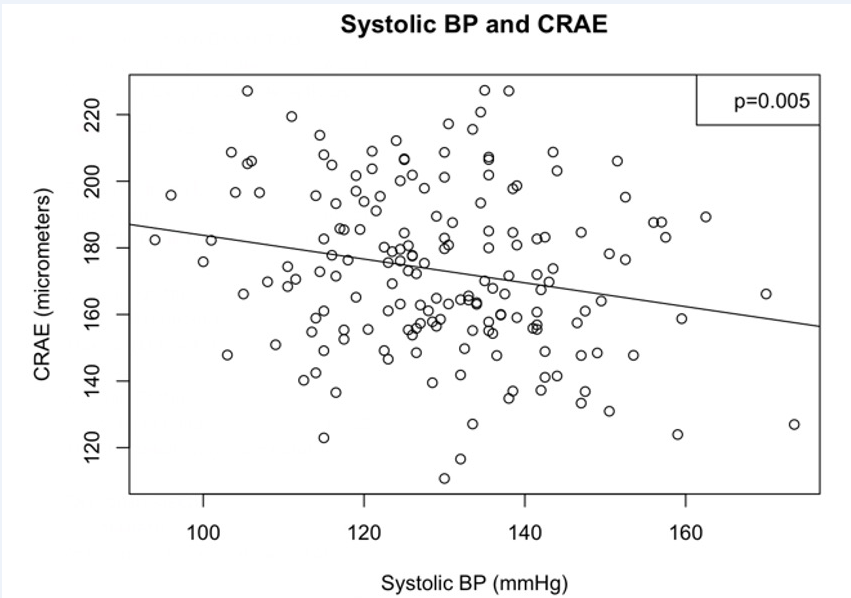

Supplement: Supplemental Digital Content [file jhype-42-1039-s001.doc]
